# Internal Medicine and Internal Medicine-Pediatrics Residency Program Website Traffic Patterns Across Two Institutions and Five Electronic Residency Application Service (ERAS) Cycles

**DOI:** 10.7759/cureus.64142

**Published:** 2024-07-09

**Authors:** Tzeidel Brown Eichenberg, Paul Fallaha, Daniel Matassa, Brent Parris, Stefanie Brown, Jonathan L Tolentino, Kristin Wong

**Affiliations:** 1 Internal Medicine-Pediatrics, Rutgers University New Jersey Medical School, Newark, USA; 2 Internal Medicine, Rutgers University New Jersey Medical School, Newark, USA; 3 Internal Medicine and Pediatrics, University of Miami Miller School of Medicine, Miami, USA; 4 Internal Medicine and Pediatrics, University of Miami, Miami, USA

**Keywords:** internal medicine-pediatrics, general internal medicine, med peds, residency applicant, residency website, residency program website design, residency recruitment, electronic residency applications, medical residency, graduate medical education (gme)

## Abstract

Background

Since the start of the COVID-19 pandemic in Spring 2020, medical residency program recruitment has become increasingly web-based due to the transition to virtual interviews. Although social media use by residency programs has soared during this time, applicant surveys show that official program websites remain the most used online resource. According to survey-based studies, the content that applicants favor on program websites tends to mirror their priorities in choosing programs. However, it is unclear whether applicant-reported priorities in program choice and website content truly drive program website traffic. In this analysis, we will elucidate website traffic patterns from two Internal Medicine residency programs and two Internal Medicine-Pediatrics programs, both in terms of the thematic content of high-traffic pages and changes in traffic patterns throughout the Electronic Residency Application Service (ERAS) application cycle. We will provide novel, data-driven guidance to residency program leadership regarding website content.

Methodology

For each of the four programs included in the analysis, total pageviews on a monthly basis were obtained for the annual ERAS application cycles taking place from 2017 through 2022. For one Internal Medicine program, data was only available for its current website from 2020 to 2022. The mean monthly pageviews were calculated for each page within each website. The total site traffic trends across each year and within high-traffic months were totaled as well.

Results

As expected, the highest traffic period for all programs in all years was the days before the application deadline, with a secondary, smaller spike in traffic around Match Day. In general, the most popular pages for all four programs were thematically in line with the top five applicant priorities in the 2022 National Resident Matching Program applicant survey, namely, geographic location, goodness of fit, program reputation, work/life balance, and current program residents. Three of the websites featured unique content that unexpectedly proved to be as popular as the top survey-reported topics, such as pages related to a new major clinical site, a new integrated subspecialty pathway, and, most profoundly, a pipeline program for certain applicants from groups that are underrepresented in medicine. Alumni career content was also heavily trafficked across all four programs.

Conclusions

Program directors should plan twice-yearly updates to residency program websites, timed to be finished by the start of the ERAS cycle in the fall, and again just before Match Day in March. Program directors should include specific, up-to-date information about unique program features. Future research should incorporate a more diverse variety of programs, software-based page content analysis, and traffic source data.

## Introduction

In the United States, medical students apply to postgraduate residency programs to complete training in their medical specialty of interest via the Electronic Residency Application Service (ERAS) and the National Resident Matching Program’s (NRMP) algorithmic match. In 2022, the average US medical school graduate applying to Internal Medicine (IM) submitted 49 applications, and to combined Internal Medicine-Pediatrics (Med-Peds), 30 applications [1]. While residency programs have communicated program details to applicants through official websites since the early 2000s, residency program websites have taken on new importance since the transition to virtual applicant interviews due to the COVID-19 pandemic. This change was first implemented for the 2020-2021 application cycle and has likely left applicants with a need for program information in the absence of an in-person interview, which traditionally included a site visit.

While the use of social media platforms for specific program information and communication has soared, official program websites remain the most commonly utilized digital platform by applicants [2-20]. According to digital media experts and seasoned residency program directors, this persistent emphasis on web browser-based content is likely because applicants can rely on institutional websites as a source of more consistent information about the details of the program, as opposed to the more fleeting, interactive nature of social media posts [12,13].

According to the NRMP’s annual applicant survey, the five most important factors to residency applicants completing their final year at US allopathic medical schools were geographic location, goodness of fit, program reputation, work/life balance, and current program residents as they selected the programs to which they would apply [14]. Similarly, applicants report that the most vital content areas on a program’s website include information regarding program faculty, current residents, alumni careers, resident wellness initiatives, program curriculum, program facilities, and research activities [15,16].

While there appear to be thematic elements in common between applicant-reported program preferences and applicant-reported areas of focus on program websites, it was previously unclear whether web pages catering to these topics are the most trafficked pages within program websites. In this analysis, we will demonstrate trends in residency program website traffic across four residency programs at two institutions in New Jersey (NJ) and Florida (FL) from July 2017 through March 2022. By comparing site traffic patterns with the NRMP’s 2022 applicant survey results, this study intends to analyze whether topics that applicants self-report as most important to them coincide with the most trafficked pages within program websites.

We posit that website traffic will demonstrate applicants’ interest in web pages pertaining to geographic location, current residents, resident clinical experience, resident life outside of work, and program academics. This analysis will aid programs in identifying which website components should take priority in website development as they continue to develop a digital marketing strategy in an increasingly online residency application process.

## Materials and methods

All available web analytics data from the current websites for the Rutgers University New Jersey Medical School’s (NJMS) Med-Peds and IM residency programs, and the University of Miami Miller School of Medicine’s (UMiami) Med-Peds and IM residency programs were included in this analysis. The NJMS sites used an internal web analytics platform, and the UMiami sites used Squarespace sites for web design and analytics. Of note, the number of applicants to each program during the years studied is shown in Table 1.

**Table 1:** Mean annual applicants. The mean number of annual applicants for each program included in the analysis during the academic years studies. Applicants are broken down by graduates of US allopathic medical schools (US MD), graduates of US osteopathic medical schools (US DO), and graduates of medical schools outside of the US (IMG).

Program	US MD	US DO	IMG	Total
NJMS Med-Peds	132	29	77	238
NJMS IM	965	545	3200	4,710
UMiami Med-Peds	199	51	77	327
UMiami IM	2,545	728	3,158	6,431

As all four programs had unique websites with varying page names and content, consistent labels (i.e., “Residents,” “Location,” “Leadership”) were applied to each page across all four websites based upon broad page content. This enabled content-based comparison of analytics data across all websites.

Web analytics data included all daily pageviews, or instances that a page was loaded, for each individual page within each website. Both NJMS programs and the UMiami Med-Peds program had available data from July 1, 2017, to March 31, 2022. UMiami IM’s current website was created in 2020; therefore, only data from July 1, 2020, to March 31, 2002, was included. Daily pageviews were summed to obtain monthly pageview data for each page. Mean monthly pageviews, as well as a 95% confidence interval, for each page was calculated across all academic years (AYs) within each of the four programs’ sites. Descriptive statistics, including mean and standard deviation, and 95% confidence intervals were calculated using Microsoft Excel’s Analysis ToolPak and the Social Sciences Statistics website’s Z-score-based single-sample confidence interval calculator, respectively. The latter tool calculated a 95% population mean interval using the equation population mean = sample mean ± Z statistic × (standard error).

Total monthly pageviews to all pages within each website were summed throughout each individual annual application cycle. Additionally, total daily pageviews to all pages within each website were summed during the most highly trafficked months of each annual application cycle, i.e., September-October and March of each year.

## Results

**Figure 1:**
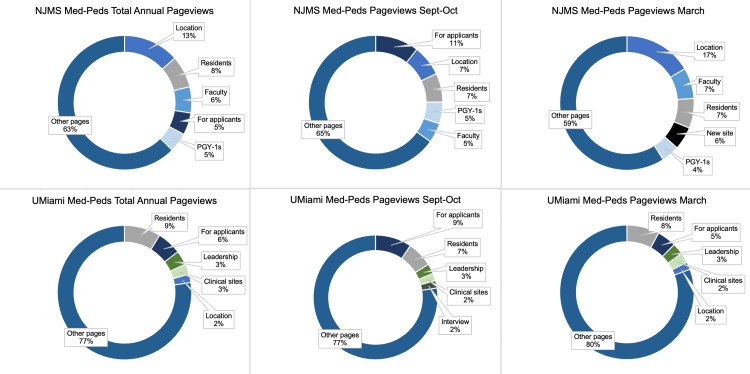
Med-Peds programs: mean pageviews by page topic. Mean pageviews by topic are shown for total annual pageviews, pageviews during September and October, and pageviews during March across all years studied. The top five most popular pages for each period are shown. PGY-1’s denotes current first-year residents.

**Figure 2:**
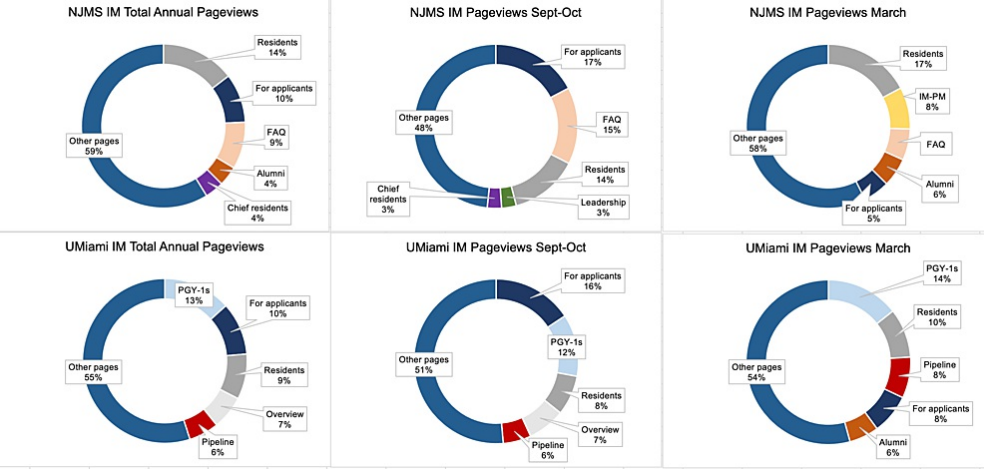
IM programs: mean pageviews by page topic. Mean pageviews by topic are shown for total annual pageviews, pageviews during September and October, and pageviews during March across all years studied. The top five most popular pages for each period are highlighted. FAQ is frequently asked questions; PGY-1’s denotes current first-year residents; IM-PM is the combined Internal Medicine-Preventative Medicine program.

The most popular pages across four programs included “Residents,” “For applicants,” “Location,” “Faculty,” “Alumni,” “Leadership,” and pages featuring the program’s current first-year residents, or “postgraduate year 1’s” (PGY-1) (Table 2, Figures 1, 2). For all programs, the five most trafficked pages differed between the Fall and Spring (Figures 1, 2).

**Table 2:** Mean annual pageviews by page topic and residency program. All data for study period 6/30/17 to 3/31/22 unless otherwise denoted. Pages accounting for 1% or less of annual pageviews are excluded. Topics that drew <1% of annual pageviews included pages displaying categorical IM and Pediatrics program leadership (for Med-Peds programs), program coordinator(s) biography, community service, photo gallery, interview day information, FAQ (for some programs), morning lecture highlights, resident publications and presentations, directions, and internal quality improvement. *: Alumni fellowship match lists >3 years old. **: The UMiami programs listed benefits information on their “For Applicants” page. ***: Notable features included pages featuring training in transitional care (for Med-Peds programs only), curriculum innovation, unique rotation experiences, and plans for future program improvement. å: The UMiami Med-Peds website included individual biography pages for all residents and recent alumni, which were omitted from this table for simplicity and privacy. These pages drew a range of 13-345 mean annual pageviews. a: Only present on the website during the academic years 21-22. b: Only present on the website during the academic year 22. c: Only present on the website during the academic years 20-22.

	Mean annual pageviews (95% CI)	Mean annual pageviews (95% CI)	Mean annual pageviews (95% CI)	Mean annual pageviews (95% CI)
Page type	NJMS IM	NJMS MP	MIA IM	MIA MP
Homepage	15,752 (13,928-17,576)	2,480 (1,757-3,203)	23,811 (18,848-28,774)	4,084 (3,341-4,827)
Residents^å^	10,809 (9,698-11,920)	1,427 (1,207-1,646)		1,739 (1,153-2,353)
PGY-1		866 (641-1,091)	13,604 (11,464-15,744)	
PGY-2		755 (560-949)	6,909 (5,730-8,088)	
PGY-3		651 (524-779)	6,811 (5,542-8,081)	
PGY-4		732 (478-985)		
For applicants	7,159 (6,433-7,886)	986 (862-1,110)	10,574 (9,389-11,759)	1,159 (659-1,660)
Faculty	1,385 (1,173-1,597)	1,162 (913-1,412)		394 (247-541)
Alumni	2,905 (2,633-3,177)	732 (532-932)	4,793 (3,483-6,103)	264 (225-303)
Archived match lists*			1,320 (458-2,182)	
Chief residents	2,751 (2185-3,317)			381 (222-541)^c^
Leadership	2,485 (2,133-2,837)	370 (363-376)^a^	3,452 (2,485-4,419)	683 (907-1411)
Program coordinators			1,332 (882-1782)	
Dept Chair			1,200 (928-1,472)	
Overview	1,375 (1,134-1,615)^a^	557 (366-748)	6,769 (5,784-7,754)	380 (341-419)
Location	948 (819-1,078)	2,185 (2,117-2,253)	1,214 (983-1,446)	400 (352-448)
FAQ	6,880 (6,079-7,681)		500 (0-1,027)	
Program contact info		307 (148-466)	1,564 (1,177-1,951)	246 (148- 345)^c^
Clinical sites	1,310 (1,085-1,535)	211 (78-343)	1,197 (983-1,446)	500 (168-626)
University Hospital^a^		261 (153-369)		
Children’s Hospital^a^		314 (220-408)		
Veteran’s Affairs Hospital^a^		161 (100-222)		
New Community Affiliate^b^		568 (n = 1)		
Continuity clinic		313 (255-371)		215 (117-173)
Sample schedule			2,280 (2,485-4,419)	235 (210-260)
Pipeline program			6,000 (2,947-9,053)	
Tracks/Subspecialty			2,264 (1,797-2,731)	
Preventative Medicine^b^	973 (n = 1)			
Medical education track	745 (435-1,054)			
Urban health track	741 (528-953)			250 (206-294)^c^
Global health track				309 (244-374)
Available fellowships	1,982 (1,679- 2,284)			
Benefits**	1,541 (1,289 - 1,793)	384 (337- 432)		
Diversity, equity, and inclusion		353 (336-369)^a^	1,124 (1,024-1,224)	264 (204.41, 323.59)^c^
Scholarly activity	725 (498-953)			239 (224-254)^c^
Wellness	708 (644-771)			357 (264-450)^c^
Resident awards	1,036 (840-1,232)			
Language training				288 (248-328)
Notable features***	806 (715-898)	173 (144-201)		260 (208-230)
Applicant webinars			2,165 (1,863-2,467)	

For the Rutgers programs, analysis of the most popular pages during the month of March showed that two newly added pages during AY’s 2021 and 2022, a page regarding a new integrated IM-Preventative Medicine (IM-PM) for the IM program, and a page regarding a new major clinical site for the Med-Peds program were some the of sites’ most popular pages (Figures 1, 2). Additionally, UMiami Med-Peds’ site showed higher traffic toward its “Interview day” page in the Fall, while its “Location” page outcompeted interview day information during March (Figure 1). UMiami IM’s site showed consistently high traffic to a page regarding a pipeline program throughout the two years of studies, as well as higher traffic to its “Alumni” page in March (Figure 2).

**Figure 3:**
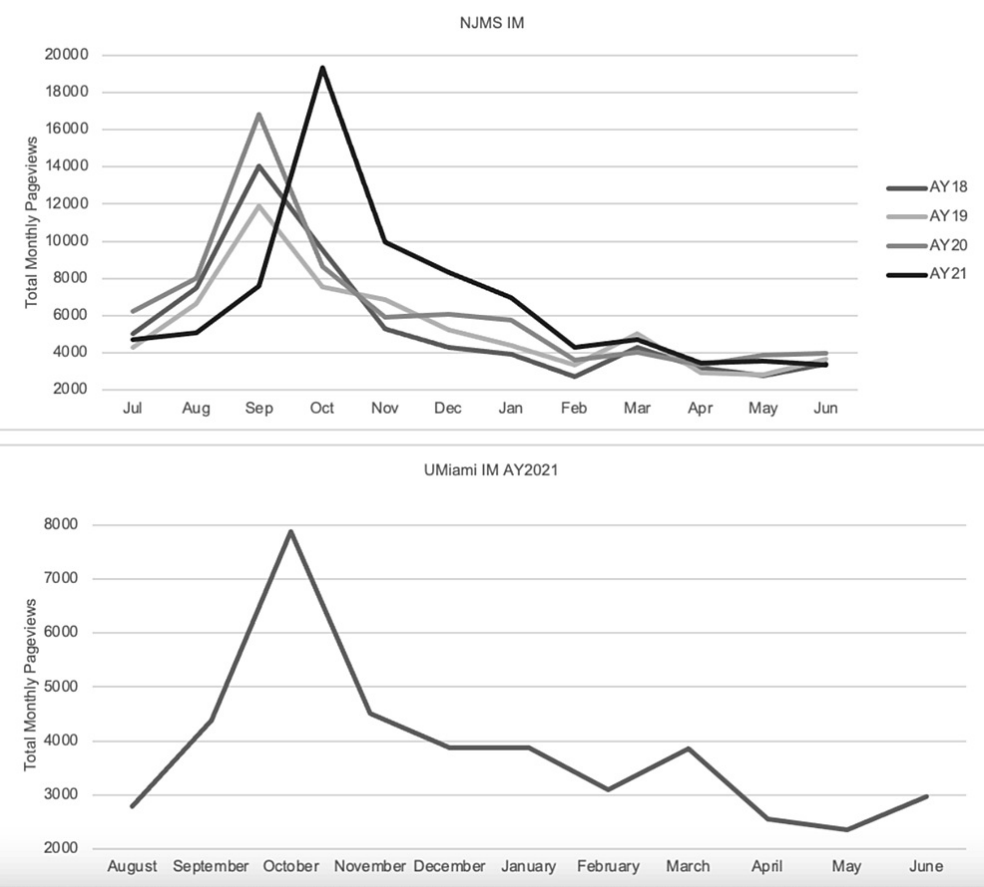
Internal Medicine programs: total monthly pageviews by year. Total monthly pageviews are shown for both Internal Medicine programs studied by academic year for full academic years with available data.

Peak traffic for all programs in all years occurred during the Fall months of September-October (Figure 3). There was a second, smaller surge in traffic in March of each year for all programs (Figure 3).

## Discussion

While many of the most trafficked sites did reflect the priorities reported by applicants in prior survey-based analyses, such as topics related to current residents, program location, and life outside of work, three unexpected topics received heavy traffic with informative seasonal variation. First, for both IM program websites, pages with information on the career trajectory of alumni were among the most heavily trafficked sites, especially during the month of March. This finding underscores the fact that, while the bulk of traffic is likely current applicants, an important audience for program leadership to consider is recently matched, new interns engaging in career planning. This is supported by the seasonal variation in website traffic. The days leading up to the application deadline in mid-September showed by far the highest traffic of any period across all four programs. In AY21 the application deadline was postponed, and its associated surge in traffic was similarly delayed that year. All four websites across all years analyzed showed a second, smaller surge in mid-March, around Match Day.

Second, both NJMS program websites added new web pages mid-cycle in AY22 that went on to surge in popularity the following March, both becoming top pages in the month of March. For Rutgers Med-Peds, the new page detailed a new community site that had recently merged resident clinical and academic activities with NJMS-affiliated training programs in both the Pediatrics and Internal Medicine departments. For IM, the popular new page detailed its new integrated IM/Preventative Medicine subspecialty program.

Third, a “Pipeline” page, unique to only UMiami IM, proved to be a major draw of traffic. This page detailed a program through which citizens of Latin American or Caribbean nations can apply to positions within the residency program reserved only for such applicants. Such programs are usually termed “pipeline” programs and serve to foster the careers of physicians who belong to ethnic or racial groups that are underrepresented in medicine (UIM), namely, Black and Latinx people [20,21].

The major limitations of our analysis pertain to the generalization of conclusions nationally, data harmonization, and lack of available traffic source data. Our analysis included data from residency program websites originating only from urban centers on the East Coast. We included no data from surgical specialty programs or subspecialty fellowship programs. In general, prior similar studies have included all programs or applicants nationally within a particular specialty or subspecialty without interspecialty analysis. With regards to data harmonization, individual researchers assigned consistent labels to pages across websites via personal review of site content with very little set methodology, as opposed to a more objective coding scheme. The most recent descriptive studies of residency website content have used such software. Lastly, traffic source data was not included due to inconsistent availability across the programs. As mentioned above, the major strength of our analysis is that it is the first published analysis of applicant interaction with residency program websites that uses website traffic data, as opposed to survey-based data.

## Conclusions

This analysis supports the following major takeaways for residency program leadership. Programs should expect large surges of traffic each fall and each Match Day and might consider twice annual website updates in advance of these periods, catering to both prospective residents and newly matched residents. In formulating website updates, leadership should consider that applicants remain interested in program updates throughout the ERAS cycle and will flock to pages detailing current program happenings or recent changes. Lastly, there is robust interest in pipeline programs for physicians from UIM groups, and easily accessible information about such programs may even draw applicants to a residency program that they may not have considered otherwise.

Future analysis of residency program website traffic should seek to improve upon the limitations of our analysis. Further study of web analytics from websites of programs with a greater geographic spread and diversity, as well as surgical specialty programs and subspecialty fellowship programs is needed to provide guidance on web design across all of graduate medical education. Given that there is inevitable heterogeneity among program websites, a scoping study of website content should use thematic content analysis software, or a similarly systematic approach, to review page content to harmonize web analytics data across programs more precisely. Additionally, traffic to residency program websites may originate from a variety of sources, including social media, search engines, and elsewhere on the institution’s website. Analysis of website traffic sources in the future will help program leadership optimize website viewership by increasing our understanding of how applicants seek out program websites, and how websites could be made more accessible to applicants.
